# An explainable AI-based blood cell classification using optimized convolutional neural network

**DOI:** 10.1016/j.jpi.2024.100389

**Published:** 2024-07-02

**Authors:** Oahidul Islam, Md Assaduzzaman, Md Zahid Hasan

**Affiliations:** a Dept. of EEE, Daffodil International University, Dhaka, Bangladesh; b Health Informatics Research Laboratory (HIRL), Dept. of CSE, Daffodil International University, Dhaka, Bangladesh

**Keywords:** White blood cells, Optimized CNN, Transfer learning, Explainable AI, SHAP, LIME, GRAD- CAM

## Abstract

White blood cells (WBCs) are a vital component of the immune system. The efficient and precise classification of WBCs is crucial for medical professionals to diagnose diseases accurately. This study presents an enhanced convolutional neural network (CNN) for detecting blood cells with the help of various image pre-processing techniques. Various image pre-processing techniques, such as padding, thresholding, erosion, dilation, and masking, are utilized to minimize noise and improve feature enhancement. Additionally, performance is further enhanced by experimenting with various architectural structures and hyperparameters to optimize the proposed model. A comparative evaluation is conducted to compare the performance of the proposed model with three transfer learning models, including Inception V3, MobileNetV2, and DenseNet201.The results indicate that the proposed model outperforms existing models, achieving a testing accuracy of 99.12%, precision of 99%, and F1-score of 99%. In addition, We utilized SHAP (Shapley Additive explanations) and LIME (Local Interpretable Model-agnostic Explanations) techniques in our study to improve the interpretability of the proposed model, providing valuable insights into how the model makes decisions. Furthermore, the proposed model has been further explained using the Grad-CAM and Grad-CAM++ techniques, which is a class-discriminative localization approach, to improve trust and transparency. Grad-CAM++ performed slightly better than Grad-CAM in identifying the predicted area's location. Finally, the most efficient model has been integrated into an end-to-end (E2E) system, accessible through both web and Android platforms for medical professionals to classify blood cell.

## Introduction

Blood, an essential bodily fluid, consists of many cells that perform critical functions in preserving well-being and combating illnesses. One of the key elements of blood testing is identifying white blood cells (WBCs). We can identify whether a disease exists, what kind of disease it is, and how severe it is by counting, comparing, and analyzing the different types of WBCs in the blood.^1^ Therefore, accurately classifying WBCs is crucial for diagnosing and surveilling a wide range of medical conditions, including infectious diseases and chronic processes, e.g., leukemia, inflammation, malnutrition, etc.^2^ Although medical technology has made significant progress, the conventional methods used for blood cell analysis can be lengthy and susceptible to human mistakes. They may not provide the level of precision required for early disease detection. Deep learning (DL), specifically convolutional neural networks (CNNs), has become a formidable tool in medical image analysis.^3^, ^4^, ^5^ It can potentially transform how we identify and treat blood-related disorders significantly.

Although CNNs have made significant progress in enhancing the accuracy and efficiency of blood cell classification, their “black-box” nature presents barriers to clinical adoption. One major obstacle is the incapacity of medical professionals to comprehend and have faith in these models' decision-making processes. Furthermore, current models compromise between interpretability and accuracy, requiring a solution that improves both aspects without compromising performance. This research aims to develop an optimal CNN model for the classification of blood cells that achieves high accuracy and is also interpretable using explainable AI (XAI). This research focuses on addressing a crucial gap in the use of DL for medical diagnostics by focusing on the interpretability of CNN-based blood cell classification models. This study contributes to the technical advancement of DL models in healthcare by integrating XAI techniques like SHAP (Shapley Additive explanations) and LIME (Local Interpretable Model-agnostic Explanations), as well as Gradient-Weighted Class Activation Mapping (Grad-CAM) and Grad-CAM++ for interpretability. Additionally, it helps bridge the trust gap between AI technologies and healthcare professionals. The findings are anticipated to support the clinical implementation of AI in blood diagnostics, allowing quicker, more precise, and transparent processes of decision-making. This method can significantly improve patients' health by enabling the early detection of diseases through a more precise analysis of blood cell abnormalities.

**Fig. 1:**
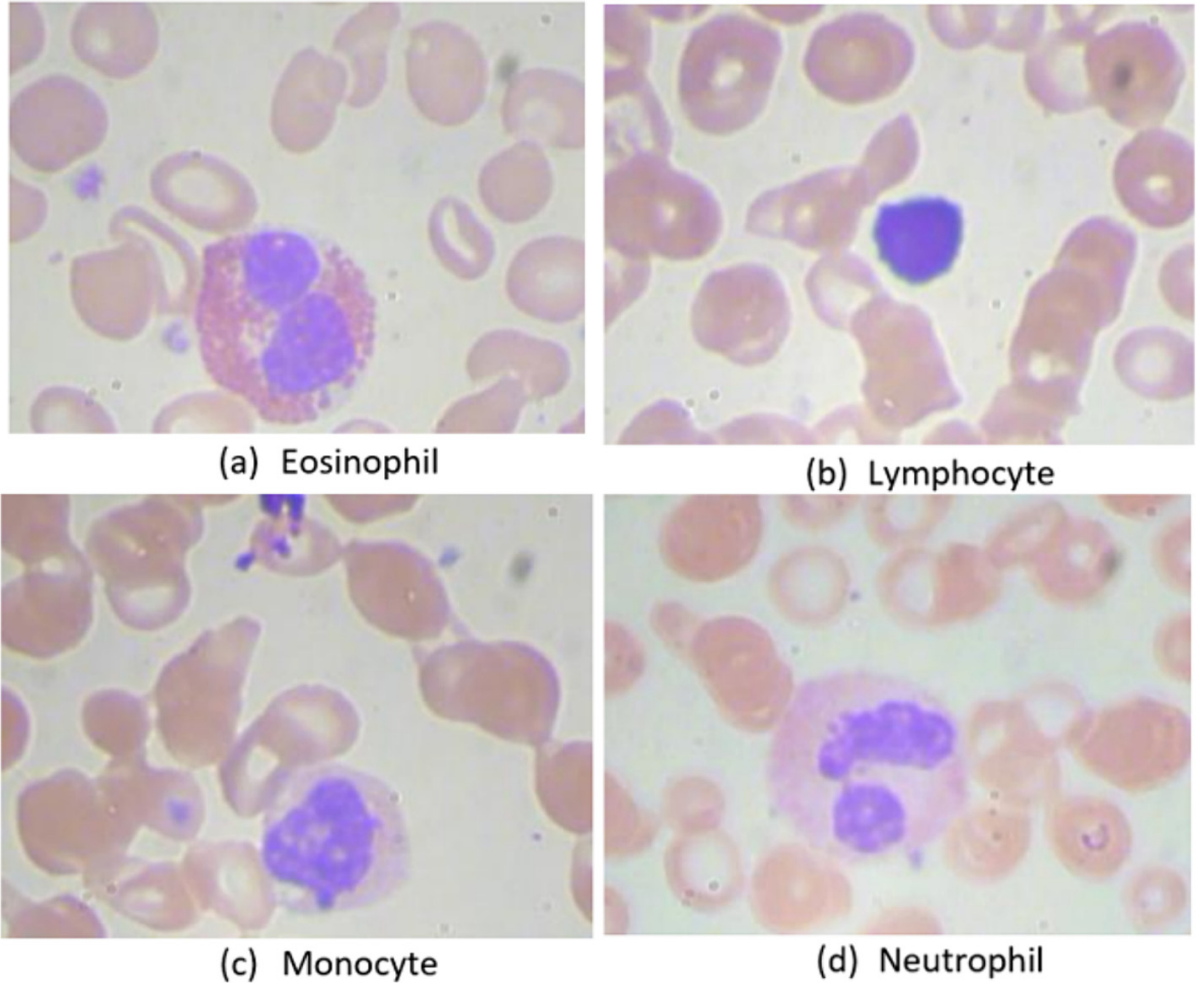
Subtype of white blood cell images.

We utilize a dual approach in our research. Our primary objective is to enhance the performance of a CNN model for blood cell classification by prioritizing accuracy. This study used a range of models based on transfer learning for CNNs and a custom CNN model to identify blood cell subtypes into four distinct groups. The performance of these models was thoroughly analyzed and compared. Additionally, we have incorporated SHAP and LIME, which can improve the model's transparency and enable medical professionals to understand its decision-making process. The methodology section describes image pre-processing, model architecture, implementation of SHAP and LIME, Grad CAM, along with Grad CAM++ for interpretability, and the implementation of the models. Fig. 1 depicts the subtype of WBCs.

The primary contributions of our study are as follows:

1. Applied various image pre-processing techniques to prioritize relevant regions and eliminate unnecessary background. This resulted in enhanced precision and scalability in classification, as well as improved computational efficiency for faster training and inference.
2. Utilized LIME and SHAP, two widely used XAI approaches, to generate saliency maps and identify the most significant features influencing the model's decision.
3. Utilized Grad-CAM++ and Grad-CAM to further enhance the interpretability of our proposed approach by generating heatmaps that increased its transparency and provided insights for better understanding.
4. Developed a real-time application and interactive web-based tools to aid medical professionals in the early identification of blood cells.

The paper begins with a review of related works on blood cell classification using deep learning techniques. Following this, it outlines our proposed architecture and presents our experimental findings in the experimental results and analysis section.

## Related works

Recent work on blood cell categorization with DL algorithms has made major advances. Zhu et al.^6^ introduced DLBCNet, a network for blood cell multi-classification, which replaced earlier models with BCGAN. They employed ResNet50 as the backbone and Ensemble Transfer Learning models to boost performance. Mosabbir et al.^7^ use the VGG16 (Retrained), VGG19 (Retrained), and DenseNet201 (Retrained) models to generate the adaptive-weighted mean. The morphological categorization of abnormal WBCs, including immature WBCs and atypical lymphocytes, as shown in peripheral blood smear images in acute myeloid leukemia (AML), is covered by Tausneem et al.^8^ The authors created a categorization algorithm for abnormal AML WBCs based on these cells' unique characteristics. Create a hybrid GT-DCAE WBC augmentation model using a deep convolutional with autoencoder (DCAE) and a geometric transformation as (GT) model. Sharma et al.^9^ employ the DenseNet121 classifier. This model uses pre-processing and data augmentation approaches for normalization. Zolfaghari et al.^10^ identify leukemia via detection and categorization. To attain the best performance metrics, the author also suggested offering models for detecting and classifying acute leukemia and WBCs that combine SVM and CNN classifiers in their classification phase. By examining the microscopic images of blood samples, Maryam et al.^11^ suggested a novel DL algorithm variation to identify leukemia illness. Also, the squeeze-and-excitation process' incorporation improves the ability to distinguish between leukemic and normal cells' features, helps to selectively reveal informative features of leukemia cells while hiding less-important ones, and increases the DL algorithm's ability to represent features. It has been noted that the suggested model performs exceedingly well in accurately identifying people with leukemia. Neeraj et al.^12^ provide a technique for automatically classifying blood cells, and the suggested technique may distinguish between granular and non-granular. By generating a heatmap image, Islam et al.^13^ used the Grad-CAM technique to identify which parts of an image the model that was suggested paid a great deal of focus on contiguous to the remaining parts, and it proposes a multi-headed attention-based transformer model to identify the malaria parasite from blood cell images. Meenakshi et al.^14^ proposed a powerful CNN algorithm for WBC count detection based on Deep Features. Erdal Başaran's^15^ study introduces an innovative diagnostic model that leverages the power of CNNs, LIME, and mRMR techniques to enhance the accuracy of identifying different WBC types. TWO-DCNN is a novel technique to WBC classification that addresses the drawbacks of traditional ML algorithms, according to Yao et al.^16^ The work emphasizes TWO-DCNN's potential as a substitute approach for clinical applications, highlighting its ability to advance the area of automated diagnostics and medical image processing. Yao et al.^17^ introduce a novel WBC classification method that includes object detection and identification in the same phase. Their results demonstrate the effective use of the Faster R-CNN and YOLOv4 models, which provide greater real-time diagnostic capabilities for high-grade patient care. Fuhad et al.,^18^ using microscopic blood smear images as a starting point, suggest a totally automated CNN-based model for diagnosing malaria and identifying malarial parasites from microscopic images. Laith et al.^19^ presents light deep learning algorithms that categorize erythrocyte into three classes: circular (normal), elongated (sickle cells), and other blood components. The transfer learning approach is used to address this problem and improve performance. Baydill et al.^20^ offer capsule networks as a strong and effective option for analyzing medical data, especially when dealing with small sample sizes. Regional CNNs (R-CNNs) have been utilized as a technique to categorize images by Huseyin Kutlu et al.^21^ Also evaluated with complete learning and transfer learning are the architectures AlexNet, VGG16, GoogLeNet, and ResNet50. Deepak et al.^22^ use a method called Optimized Binary Bat to classify various kinds of leukocytes. Four distinct classifiers, K-nearest neighbors (KNN), logistic regression, random forest, and decision tree, are used to construct the suggested technique. Shahin et al.^23^ provide a ground-breaking technique for identifying WBCs that is based on DL concepts. The work contributes to the larger effort of automating health diagnostics and enhances the area of medical image analysis while providing a potential answer to WBC detection. Asghar et al.^24^ examined in medical image analysis the application of machine learning (ML) as well as DL algorithms for WBC categorization. Examining 136 papers between 2006 and 2023, the analysis highlights field difficulties as well as developments. Important obstacles include the accessibility of suitable datasets, the requirement of improved medical education for investigators, and the acceptance of sophisticated DL networks such as GANs and R-CNN variations. The research underlines how crucial these models are for raising WBC categorization diagnosis accuracy as well as for other medical imaging uses.

Identify blood cells and perform medical research by customizing and training various ML and DL models on specific datasets. These models include various deep neural networks, including ResNet50, VGG16, VGG19, DenseNet201, DenseNet121, AlexNet, and GoogleNet also include a variety of cutting-edge architectures, including YOLOv4.^6^^,^^7^^,^^9^^,^^18^^,^^21^ Author Mosabbir et al.^7^ adopted an information supplementation technique to increase the quantity of data and alleviate the model's overfitting difficulty. The proposed ensemble learning model performs the best overall, correctly distinguishing parasitized and uninfected cells with a 97.92% accuracy. Also, finding focused areas in blood cells performing GRAD-CAM, LIME, and mRMR techniques enhanced identification accuracy.^13^^,^^15^

Many authors used image pre-processing techniques to guarantee that the input data used for ML models was high quality, consistent, and contained crucial information for precise analysis and classification.^8^, ^9^, ^10^^,^^19^ It contributes to tackling the problems inherent in real-world picture data, resulting in more robust and dependable models. The study of Baydill et al.^20^ establishes capsule networks as a potentially game-changing method for precise WBC classification, revolutionizing the field of computer-aided diagnostic systems and advancing medical image analysis without the need for extensive data pre-processing or augmentation.

Various dataset sizes are used to execute blood cell identification operations, reflecting the heterogeneity in accessibility and consumption in clinical image analysis. Mosabbir et al.^7^ used 27,558 image data from two classes and found an accuracy of 97.52%, whereas Yao et al.^17^ used only 364 image data from four classes and found a 96.25% accuracy. Using the same dataset^6^^,^^9^^,^^14^^,^^15^ to find different types of accuracy among them, Sarang Sharma et al.^9^ employ the DenseNet121 classifier. This model uses pre-processing and data augmentation approaches for normalization. The authors state that their model has a sensitivity of 98.85%, a specificity of 99.61%, and an accuracy of 98.84%. Meenakshi et al.^14^ suggested an approach that demonstrated amazing accuracy with 0.97 by unique feature extraction, hybrid feature selection, advanced classification algorithms, and precision in categorizing diversity. This study contributes to automated diagnostics and medical image analysis by providing a useful tool for detecting blood disorders connected to WBC anomalies. Erdal Başaran's^15^ suggested algorithm for determining WBCs scored 95.88%. This research not only addresses the limitations of manual interpretation but also advances the field of medical image analysis with promising implications for automated disease detection and diagnosis. The limited size of the dataset uses^16^^,^^17^^,^^20^^,^^21^^,^^23^ to find accuracy of 91.6%, 96.86%, 96.86%, and 96.1%, respectively. The author^23^ successfully overcame the difficulties of limited dataset classification by introducing the high-performance WBCsNet as a pre-trained network through the strength of deep CNNs. In Table 1, we have discussed limitations of relevant research.

**Table 1:** Limitations of existing related works.

Paper by author	Research limitations
Zhu et al.^6^	1. No segmentation, 2. Low accuracy
Bhuiyan et al.^7^	1. Dependability on a particular dataset 2. Possible overfitting
Elhassan et al.^8^	1. Need for thorough benchmarking due to the lack of balanced data, 2. Insufficient attention to unusual WBCs, potential generalizability issues 3. Implementation complexity.
Sharma et al.^9^	1. Specific dataset 2. Do not perform other pre-processing steps 3. Lack of statistical validation 4. Limited optimization technique
Zolfaghari et al.^10^	1. Focus on existing method, no new experiment 2. Not focus on pre-processing steps that impact on overall performance of model 3. Theoretical nature
Bukhari et al.^11^	1. Limited dataset 2. Specific types 3. No comparison with other models
Baghel et al.^12^	1. Limited dataset 2. Complex model architecture 3. Limited performance metrics 4. Lack of real-world validation
Islam et al.^13^	1. Specific to malaria dataset 2. No real-world validation of model 3. Limited decision-making process only used Grad-CAM 4. Not focus on image processing steps
Meenakshi et al.^14^	1. No real-world implementation 2. Absence of memory utilization
Basaran et al.^15^	1. Limited dataset 2. No feature extraction and selection 3. No comparative analysis
Yao et al.^16^	1. Network optimization needed 2. Lower size dataset 3. Single optimizer used
J.Yao et al.^17^	1. May not detect and classify small WBC target 2. Not addressed staining free WBC 3. Classification only depends on pre-processing
Fuhad et al.^18^	1. Limited image resolution 2. No examine the possible trade-offs in higher-resolution photographs between computer efficiency and possible loss of detail
Alzubaidi et al.^19^	1. Limited feature 2. Consume time 3. Binary classification only 4. No real-world implementation
Baydilli et al.^20^	1. Limited size dataset 2. No comparative analysis with other modls 3. No details information about insight challenge or limitations
Kutlu et al.^21^	1. No implementation 2. Used transfer learning only 3. No state-of-art method and comparative analysis
Gupta et al.^22^	1. Limited dataset 2. No comparison with traditional feature selecting methods 3. No validity testing 4. No benchmark testing of suggested algorithm
Shahin et al.^23^	1. Complexity of model that may occur higher training time 2. No benchmark performance is established 3. Segments the main part but other small parts may affect the result also 4. No real-life validity tests

Blood cell identification is relevant in a range of medical applications. DL methods have notable benefits in automating and enhancing blood cell identification jobs, leading to increased efficiency. Through the use of robust DL models, healthcare practitioners can augment diagnostic precision, optimize operational processes, and acquire a significant understanding of a patient's state of health.

## Proposed architecture

### System architecture

**Fig. 2:**
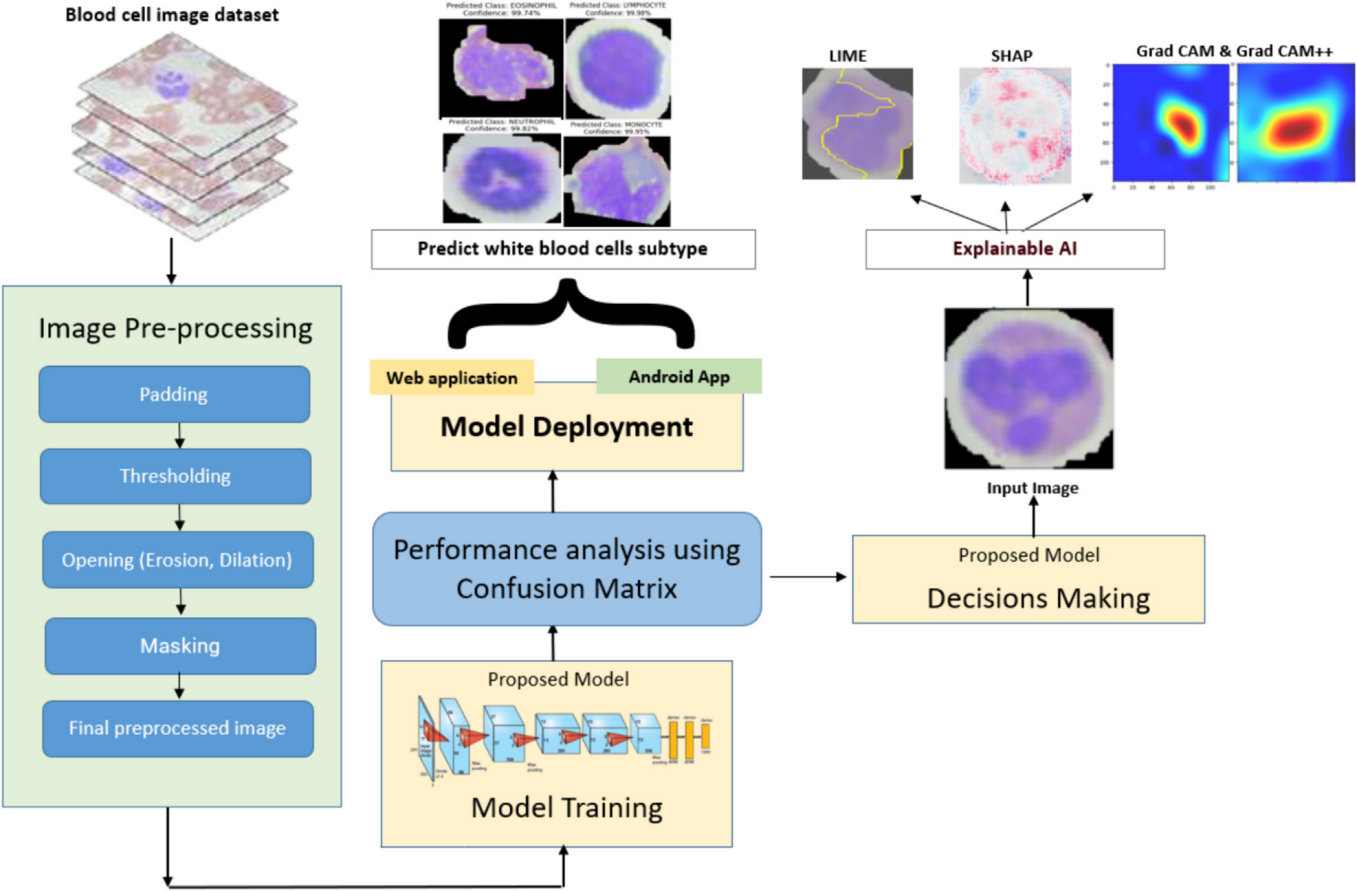
Proposed architecture to detect blood cell subtypes.

The methodology utilized to diagnose WBCs consists of four main stages: data collecting, image pre-processing, designing a proposed CNN model, and XAI. Fig. 2 provides a visual representation of the proposed approach framework. The following sections provide a detailed explanation of each phase.

### Dataset description

The study analyzed the “White Blood Cell Classification” dataset to predict WBC types from microscopic images.^25^ The dataset is organized into specific training and testing sections and has four primary categories: EOSINOPHIL, LYMPHOCYTE, MONOCYTE, and NEUTROPHIL. The dataset consists of 12,444 photos separated into validation, testing, and training sets, as indicated in Table 2. We implemented stratified sampling to ensure a well-balanced distribution across the training, testing, and validation stages. In the training folder, there are 8714 images, with each class allocated an appropriate number for training purposes. For model robustness, 15% of the training images are randomly chosen for validation with the ImageDataGenerator library. The testing dataset contains 1865 images, equally split with 1865 images for each class.

**Table 2:** Blood cell subtype dataset.

Class ID	Class name	Quantity	Training	Testing	Validation	Description
C0	EOSINOPHIL	3120	2184	468	468	The cells possess a bilobed nucleus and exhibit conspicuous reddish-orange granules inside their cytoplasm.
C1	LYMPHOCYTE	3103	2173	465	465	A distinctive large, round nucleus occupies most of the cell's volume, and there is a thin rim of pale blue cytoplasm around the nucleus.
C2	MONOCYTE	3098	2170	464	464	Larger white blood cells with a single, kidney-shaped nucleus and a pale cytoplasm.
C3	NEUTROPHIL	3123	2187	468	468	These cells have multiple lobes in their nuclei, ranging from two to five, and a neutral staining characteristic in their cytoplasm.
		Total: 12,444	Total: 8714	Total: 1865	Total 1865	

### Pre-processing steps

Image pre-processing techniques optimize computing efficiency and improve overall system performance. The dataset was pre-processed to enhance image quality and prepare it for analysis. This involved resizing images, converting them to threshold values, and padding for edge detection, erosion, and dilation.

#### Image resize

Image resizing is vital in preparing the input data for blood cell classification. Resizing assures that all images have the same dimensions, allowing them to be processed by the neural network. CNNs are built on convolution layers, which are utilized to extract characteristics.^26^ We resize images in 120×120 for each class image in the dataset. For that reason, model reliability increases. Eq. (1) represents the scaling operation.

(1) $$\left(w_{\mathrm{new}},h_{\mathrm{new}}\right)=\frac{M\left(w,h\right)}{\max\left(w,h\right)}$$

Where *w* and *h* represent width and height, respectively. *M* is the transform matrix. $w_{\mathrm{new}},h_{\mathrm{new}}$ gives the resized image.

#### Padding for excellent cell edge detection

Padding in image processing refers to adding a pixel border around the margins of an image. It enhances edge recognition and reduces data loss at image borders. Padding facilitates proper identification and segmentation of cells in blood cell analysis by keeping image edge information.^27^ For our dataset, 10 pixels will be added to all four corners of the images with a specific color-constant border. Calculate padding by using Eq. (2).

(2) $$\mathrm{conv}\_\mathrm{output}=\frac{n+\left(2*p\right)-f}{s}+1$$

In this equation, to find out conv_output, n is the input size, p stands for padding size, and f and s are filter size and stride, respectively.

#### Convert image into the threshold value

Convert an image to a threshold value using a technique to binarize the image,^28^ which means that all pixel values over a specific threshold become one color (typically white), and all pixel values below the threshold become a different color (usually black). This is frequently used for image segmentation and object detection. Applying a threshold in the context of blood cell identification can assist in separating blood cells from the background, making subsequent image processing and analysis easier. Eqs. (3), (4) show the image thresholding.

(3) $$T=T\left[x,y,p\left(x,y\right),f\left(x,y\right)\right]$$

(4) $$g\left(x,y\right)=\left\{\begin{array}{l} 1\;\text{if}\;f\left(x,y\right)>1\\ 0\;\text{if}\;f\left(x,y\right)\leq 0 \end{array}\right)$$

Here, *T* is the threshold amount. *x* and *y* are the location coordinates of the threshold's point. The coordinates $p\left(x,y\right)$ and $f\left(x,y\right)$ represent the gray level of picture pixels.^29^ The threshold image, $g\left(x,y\right)$. For thresholding, using the lower and upper bound of color range (80, 80, 180), (180, 170, 245)).

#### Erosion

We applied morphological erosion to enhance the segmentation of blood cells in the images. For effective noise reduction and cell cluster separation,^30^ utilizing a carefully chosen structural element is crucial. The erosion technique effectively reduced the images' complexity, preserving the cells' integrity and facilitating accurate extraction and analysis of their features. The erosion function in the field of mathematical morphology is usually defined in Eq. (5).

(5) g=f⊖s

#### Dilation

We utilized dilation to enhance the clarity of cell structures and connectivity. This process allowed for filling gaps and improving cell contours by using a structuring element designed for blood cells' specific features. The dilation technique improved the analysis by making cell features more visible and helped address the difficulties caused by overlapping cells and unclear boundaries.^31^ The dilatation of a representation f by a structural element s is defined in Eq. (6).

(6) g=f⊕s

#### Masking

Masking is a method for selectively emphasizing or diminishing specific areas of an image.^32^ A ‘mask’ is created by generating a binary image that matches the original image's dimensions. The mask distinguishes between pixels that require analysis (typically marked as 1 or 255 for white) and those that should be disregarded (usually marked as 0 for black). Masking is an essential pre-processing step that enables targeted and efficient analysis by separating the ROI from the rest of the image.

(7) $$R\left(x,y\right)=I\left(x,y\right)\cdot M\left(x,y\right)$$

**Fig. 3:**
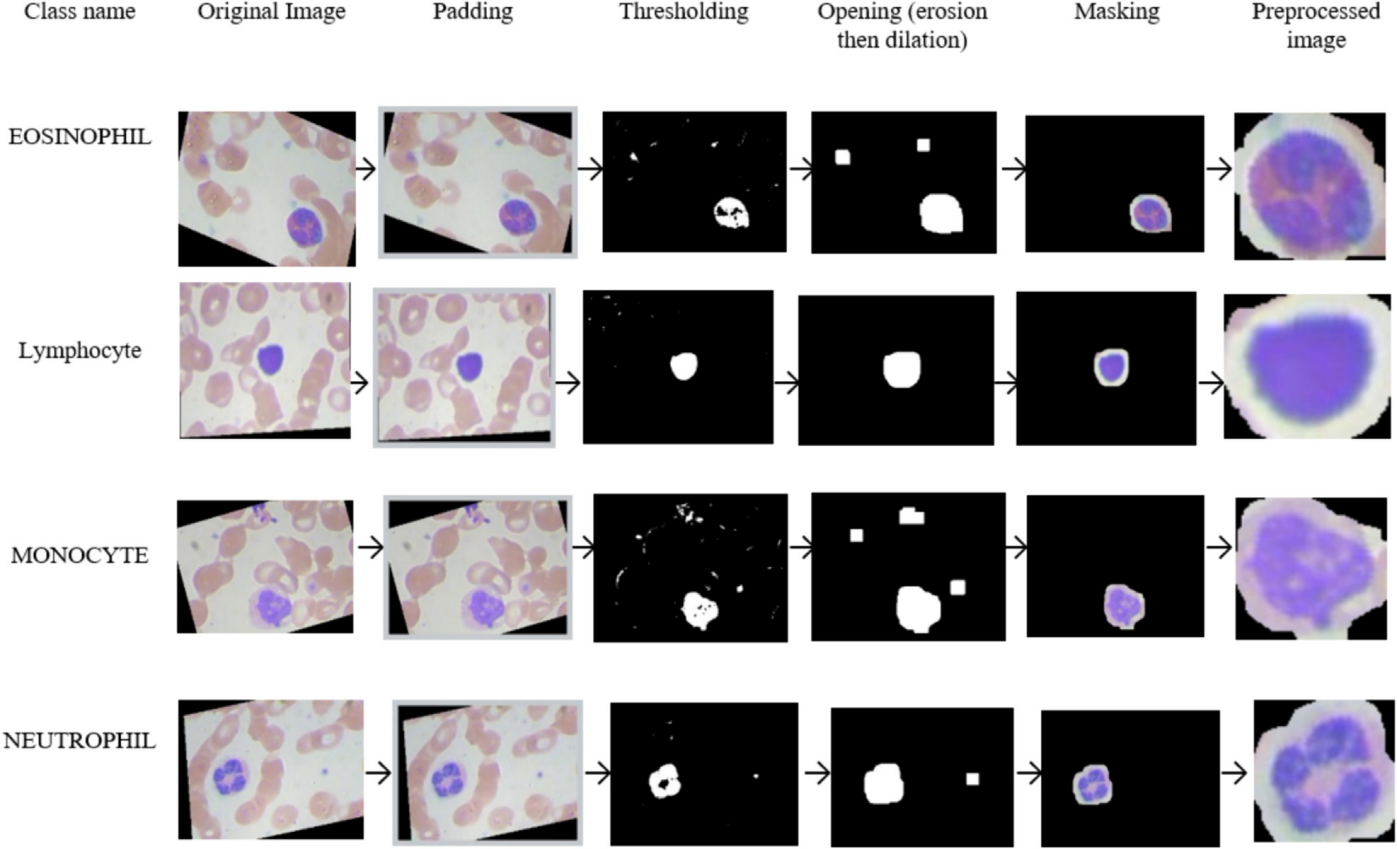
Image pre-processing steps.

Fig. 3 visualizes the entire image pre-processing operation. The pre-processing processes encompass several operations, including applying padding, thresholding to isolate the target cell, performing erosion and dilation operations, detecting the outlines of the blood cells, masking the area outside the contour, and finally extracting and resizing the blood cell image.

### Proposed model

**Fig. 4:**
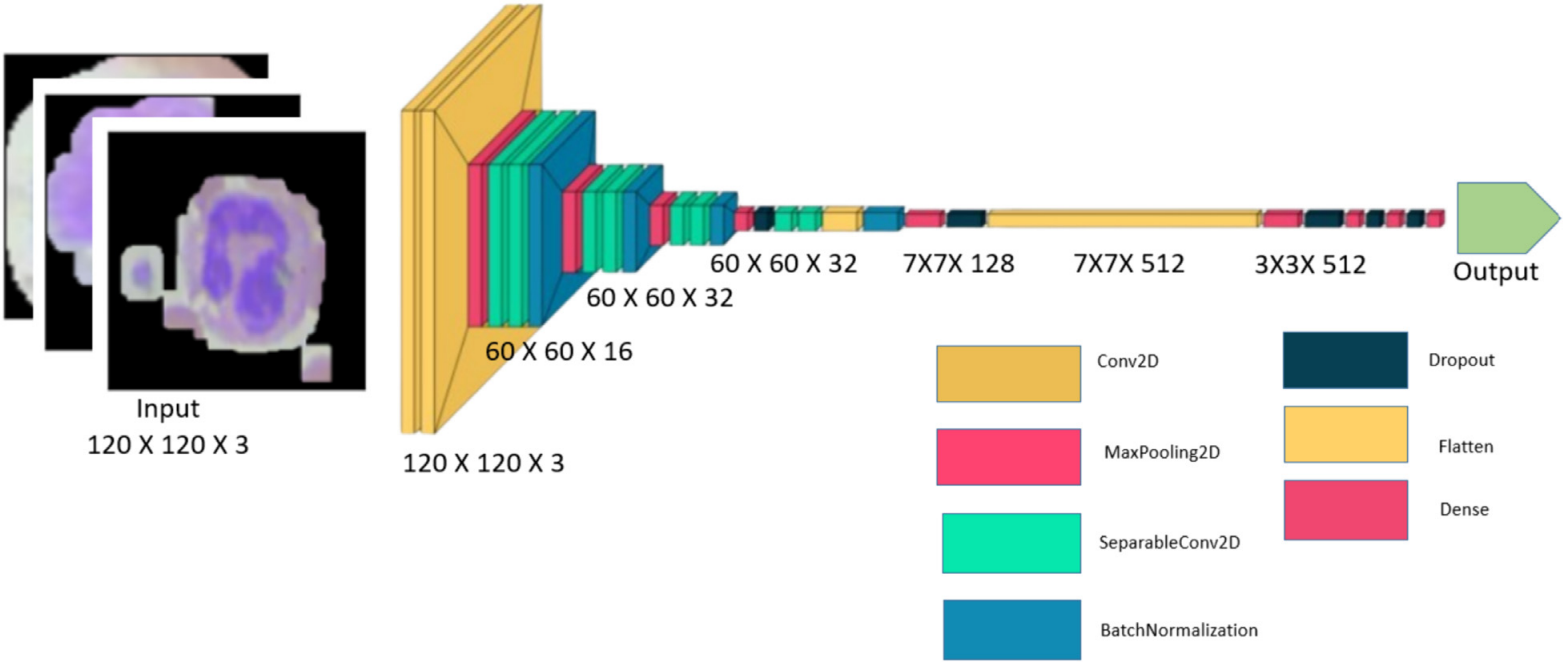
Proposed architecture for CNN.

The proposed CNN architecture comprises six convolution blocks are explained in Fig. 4. The initial block includes two convolutional layers, each with 16 filters. These are then followed by ReLU activation and a max pooling layer for downsampling. The following blocks adhere to a consistent pattern, incorporating different numbers of separable convolutional layers and filter sizes. They are complemented by batch normalization for feature normalization and max pooling for additional down sampling. In addition, the fourth and fifth blocks have a drop-out layer with a dropout rate of 0.2 to address overfitting. After the convolutional blocks, the feature maps are flattened and go through fully connected layers. These layers consist of three units: 512, 128, and 64, respectively. A hyperbolic tangent activation function follows each unit to add non-linearity. Regularization techniques, such as applying dropout layers with different rates (0.7, 0.5, and 0.3), are used to prevent overfitting in the network. The model's final layer is a dense layer with SoftMax activation. It produces probabilities for each class in the classification task, with four units corresponding to the number of classes. This architecture efficiently extracts hierarchical features from input images and accurately classifies them into distinct categories.

The Nadam optimizer is used to set up the optimizer in the suggested custom CNN architecture, which minimizes the prediction error. Adam and Nesterov Momentum are combined in NADAM, or Nesterov-accelerated Adaptive Moment Estimation in Eq. (8).^33^

(8) $$\theta_{t+1}=\theta_{t}-\frac{\eta }{\sqrt{{\hat{v}}_{t}}+\epsilon }\left(\beta_{1}{\hat{m}}_{t}+\frac{\left(1-\beta_{t}\right)g_{t}}{1-\beta_{1}^{t}}\right)$$

Compared to other optimization algorithms like Adam and SGD, which use the momentum term to speed up convergence, NADAM converges to the objective function's minimum more quickly. Advantages of this optimizer include its ease of implementation for DL professionals who want a strong optimization technique without having to create complicated code and frequently choose it. Also, this optimizer is more resilient to subpar initializations than SGD and Adam, two other optimization techniques. NADAM is a suitable optimization method for managing noisy gradients in goal function optimization, as it uses the Nesterov Momentum technique, which prevents oscillations that could hinder convergence, making it more suitable than other methods.

(9) $$g_{t}\leftarrow \nabla_{\theta_{t-1}}J_{t}\left(\theta_{t-1}\right)$$

(10) $$m_{t}\leftarrow \beta_{t}m_{t-1}+\eta g_{t}$$

In Eq. (10) for greater clarity in this type of optimizer, a set of factors *β*1, *β*2, *βt*, which correspond to steps 1, 2, …, *t* is taken into consideration in order to raise and decrease the decay factor β over time in Eq. (10).^34^

In an optimization algorithm, the learning rate is an adjustment parameter that establishes the step size at every iteration as the algorithm moves towards a loss function minimum.^35^ For this proposed custom CNN approach, the learning rate is 0.00001. Utilizing an average learning rate to ensure no local minimums are missed. An excessively high- or low-learning rate has an impact on training loss.

(11) $$\mathrm{New}\;\mathrm{Parameter}=\mathrm{Old}\;\mathrm{Parameter}-\left(\mathrm{Learning Rate}*\mathrm{Gradient}\right)$$

Here, Eq. (11) explains how a model's parameter is changed via a gradient descent-based optimization approach with each training iteration.

For this proposed custom CNN, the uniform weight initialization proposed by Xavier Glorot is now in place. The Xavier Glorot initialization for a uniform distribution determines the weights by utilizing a random selection process from a uniform distribution, with restrictions based on the quantity of input and output units inside a layer. Eq. (12) shows the weight initialization process:

(12) $$W∼U\left(-\frac{\sqrt{6}}{\sqrt{n_{\text{in}}+n_{\text{out}}}},\frac{\sqrt{6}}{\sqrt{n_{\text{in}}+n_{\text{out}}}}\right)$$

Here, in Eq. (12), w is the weight matrix, $U\;\left(a,b\right)\;$denotes a uniform distribution between a and b, *n* is the amounts of units in the layer as input, and $n_{\mathrm{out}}\;$is the number of elements in the final product layer.

This work uses categorical cross entropy to make the model aim for precise and confident predictions by penalizing confidently incorrect predictions more severely. Throughout the training, model weight adjustments are made via cross-entropy loss.^36^ The goal is to reduce the loss; a high-graded model has fewer losses. Eq. (13) defines categorical cross entropy classification:

(13) $$H\left(p,q\right)=-\sum_{x\;\text{celasses}\;}\;p\left(x\right)\log q\left(x\right)$$

Here, in Eq. (14), $p\left(x\right)\;$represents true probability distribution and log $q\left(x\right)\;$model's predicted probability distribution. The distinction between the probability distributions that are discovered and those that a classification model predicts using DL is measured by cross-entropy loss. Eq. (14) denotes the cross-entropy formula,^36^

(14) $$\mathrm{CE}=-\sum_{i}^{C}\;t_{i}\log\left(f{\left(s\right)}_{i}\right)$$

The activation function of Softmax is often applied to test scores before the computation of the CEloss.^36^ Softmax activation function in Eq. (15), where the function of activation is denoted by *f* (*s*).

(15) $$f{\left(s\right)}_{i}=\frac{e^{s_{i}}}{\sum_{j}^{C}\;\;e^{s_{j}}}$$

The proposed model runs with 30 epochs with batch size 64, which means that training time and images are batched with 64 quantities. An ablation study employed this number of batch sizes for this model. The quantity of data samples handled collectively in each optimization iteration of a model depends on the batch size during model training. Because lower batch sizes might generate more noise but may converge quicker owing to more frequent updates, larger batch sizes frequently offer computational effectiveness but may result in a less noisy gradients estimate. Balance keeping strong performance on fresh, unknown samples with fitting the training data as well as minimizing validation loss.

### Transfer learning (TL) models

#### DenseNet201

The DenseNet201 architecture utilizes densely connected blocks,^37^ allowing each layer to receive direct input from all preceding layers. This promotes feature reuse and improves gradient flow. The architecture comprises several compact blocks, each containing densely connected convolutional layers. Transition layers are used to enable downsampling through pooling and dimensionality reduction. Stable training and non-linearity are promoted by applying batch normalization and ReLU activation after each convolutional layer. The network concludes with global mean pooling, which is followed by an entire linked layer that uses SoftMax activation for multi-class classification. DenseNet201 provides a robust framework for image classification tasks thanks to its strong connectivity and effective feature propagation.

#### Inception V3

Inception V3 is a highly accurate neural network architecture designed for reliably identifying images.^38^ The network utilizes multiple inception modules incorporating parallel convolutional operations of different kernel sizes, enabling efficient feature capture at various spatial scales. The inception module comprises convolutional layers with various filter sizes and pooling operations, allowing the model to extract a wide range of distinctive features from input images. In addition, Inception V3 incorporates auxiliary classifiers to address the issue of vanishing gradients during training. In addition, it utilizes batch normalization and factorized 7 × 7 convolutions to decrease computational complexity while still achieving high performance. The architecture is highly regarded for its ability to handle various visual recognition tasks with exceptional precision efficiently.

#### MobileNetV2

MobileNetV2 is a very effective CNN design that employs depth-wise segmented convolutions and linear constraints to balance computational complexity and accuracy.^39^ The architecture comprises several inverted residual blocks, each comprising convolutional layers, batch normalization, and ReLU activation functions. In addition, MobileNetV2 utilizes shortcut connections and expansion layers to enhance information flow and feature reuse between layers. MobileNetV2 is highly suitable for real-time image classification and object detection tasks on devices with limited resources, thanks to its lightweight operations and efficient design principles.

## Experimental results and analysis

### Metrics for evaluation

DL and ML evaluation metrics are essential tools for assessing model performance to determine how well the model is.^40^ They aid in evaluating how well models do certain tasks. Predictive accuracy in classification is measured using measures including accuracy with precision, recall, and F1-score value.^40^

#### Accuracy

The proportion of accurately predicted images forecasts is called accuracy. The accuracy is expressed as follows in Eq. (16)

(16) $$\mathrm{Accuracy}=\frac{\mathrm{TP}+\mathrm{TN}}{\mathrm{TP}+\mathrm{TN}+\mathrm{FP}+\mathrm{FN}}\;$$

The accuracy score calculates a model's proportion of precise forecasts $\left(\mathrm{TP}+\mathrm{TN}\right)$ made to all guesses TP+TN+FP+FN produced. True positive, true negative, false positive, and false negative are each represents as the acronyms “TP,” “TN,” “FP,” and “FN,” respectively.

#### Precision

This metric calculates the percentage of true positives among all positive cases. Eq. (17) determines the precision:

(17) $$\mathrm{Precision}=\frac{\mathrm{TP}}{\mathrm{TP}+\mathrm{FP}}\;$$

#### Recall

By comparing the number of actual positive specimens to the total number of true-positive findings, the recall value is utilized to evaluate the accuracy of positive predictions. $\left(\mathrm{TP}+\mathrm{TN}\right)$. For calculating the recall value by Eq. (18):

(18) $$T\;P\;R=\frac{\mathrm{TP}}{\mathrm{TP}+\mathrm{FN}}$$

#### F1-score

This metric assesses the overall effectiveness of the model through its combination of recall and precision values. It is defined in Eq. (19).

(19) $$F1-\mathrm{Score}=2*\frac{\mathrm{Recall}*\mathrm{Precision}}{\mathrm{Recall}+\mathrm{Precision}}\;$$

### Result analysis

In this subsection, we have examined and detailed the performance of the applied model. The training, validation accuracy, loss graph, and confusion matrix of the models have been demonstrated. The evaluation metrics, final model accuracy, and training and validation losses were calculated to assess model performance. Additionally, other relevant studies have evaluated the performance of suggested model.

**Fig. 5:**
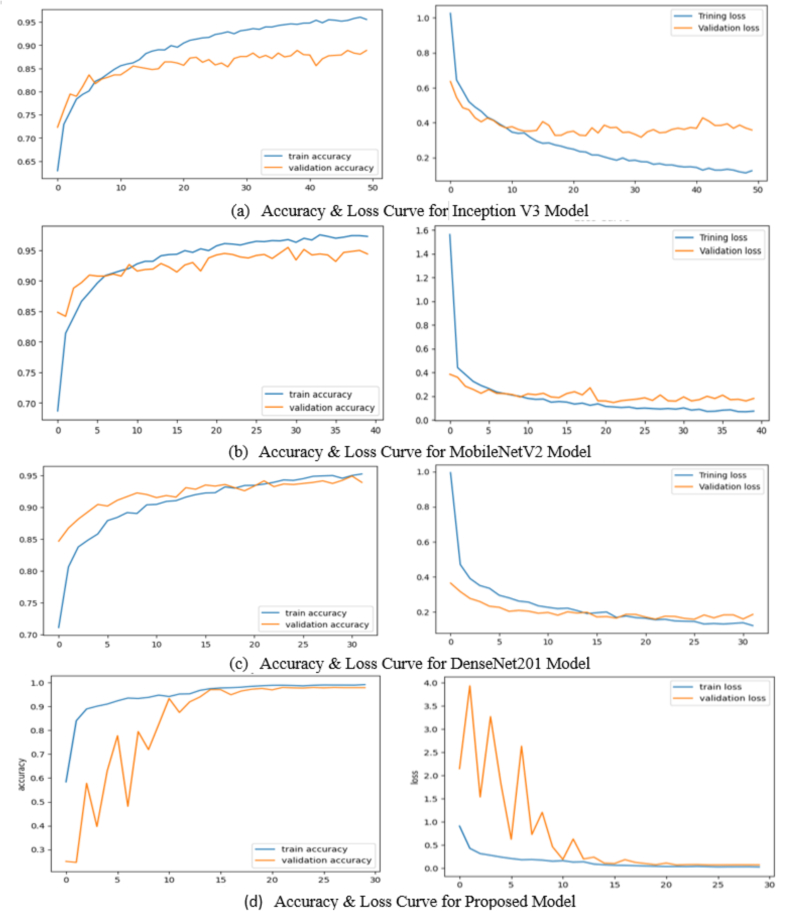
Accuracy and loss graph for Inception V3, MobileNetV2, DenseNet201, and proposed model.

Our investigation compared the performance of Inception V3, MobileNetV2, DenseNet201, and a proposed model. In Fig. 5(a), Inception V3 demonstrated a notable disparity in performance between training and validation accuracies. Because the training accuracy showed consistent improvement, the validation accuracy did not show similar progress. This disparity indicates that Inception V3 may be overfitting to the training data and unable to generalize adequately to the unseen validation data. This disparity indicates that Inception V3 may be overfitting to the training data and fails to effectively generalize to the unobserved validation data. On the other hand, from Fig. 5(b) and (c), we can observe that MobileNetV2 and DenseNet201 demonstrated better performance than InceptionV3 in training and validation metrics. However, our proposed model outperformed all other models, consistently improving training and validation accuracies with minimal disparity (in Fig. 5(d)). The exceptional performance of the proposed model highlights its effective learning and generalization abilities, establishing it as the most suitable model for the classification task.

The training parameters of the proposed architecture is shown in Table 3. Assessment criteria such as accuracy, precision, sensitivity, and F1-score are used to assess the implemented model's performance. The performance of the applied model, as determined by evaluation measures, is presented in Table 4. Inception V3 shows balance metrics with a recall, precision, and F1-score of 0.88, suggesting good but not optimal performance. MobileNetV2 demonstrates enhancements in all metrics at 0.93, indicating improved model efficiency and accuracy. DenseNet201 has significantly improved performance, achieving a high score of 0.96 across all categories, showcasing its robust ability to classify and generalize from the data accurately. The proposed model outperforms all others with outstanding scores of 0.99 in recall, precision, and F1-score, showcasing high accuracy and reliability in identifying relevant instances and an excellent balance between precision and recall. The proposed model demonstrates exceptional performance and effectiveness in the evaluated task.

**Table 3:** Lists the proposed CNN model's training parameters.

Parameter	Description
Optimization algorithm	Nadam optimizer
Input shape	(120,120,3)
Rate of learning ()	0.00001
Initialization of weight	Glorot uniform
Size of the batch	64
Number of epochs	30
Drop-out rate	0.2 or 20%
Loss function	sparse_categorical_crossentropy
Activation function (Hidden layers)	Relu
Activation function (Output layer)	SoftMax

**Table 4:** The performance of evaluation metrics in models.

Used models	Recall	Precision	F1-score
Inception V3	0.88	0.88	0.88
MobileNetV2	0.93	0.93	0.93
DenseNet201	0.96	0.96	0.96
**Proposed model**	**0.99**	**0.99**	**0.99**

Table 5 shows that the proposed model demonstrates the highest accuracy compared to the other evaluated models. It also has a competitive validation loss and relatively lower computational complexity. The proposed model showcases exceptional performance in blood cell classification tasks with a test accuracy of 99.12%, striking a harmonious balance between accuracy and computational efficiency.

**Table 5:** Model accuracy and loss throughout training and validation are assessed.

Model	Accuracy	Accuracy training	Validation	Loss training	Validation	Time complexity (seconds)	Space complexity (megabyte)
Inception V3 model	88.29%	0.995	0.888	0.025	0.3625	572.39	85.207
MobileNetV2 model	93.32%	0.997	0.954	0.015	0.1591	501.54	9.887
DenseNet201 model	95.21%	0.988	0.953	0.045	0.1635	1820.99	71.802
**Proposed model**	**99.12%**	**0.993**	**0.928**	**0.023**	**0.026**	**245.881**	**14.33**

Table 6 shows the class-wise evaluation metrics from the Inception V3, MobileNetV2, DenseNet201, and proposed model. C0, C1, C2, and C3 represent Eosinophil, Lymphocyte, Neutrophil, and Monocyte, respectively. The findings show that, compared to other models, the proposed models provide higher evaluation matrix values.

**Table 6:** The current study looks at the recall, accuracy, and F1-score of several suitable models on a class-by-class basis.

	Class name	Value of recall	Value of precision	Value of F1-score
Inception V3 model	C0	0.80	0.82	0.81
	C1	0.97	0.96	0.97
	C2	0.93	0.95	0.94
	C3	0.83	0.80	0.82
MobileNetV2 model	C0	0.91	0.86	0.88
	C1	0.99	0.99	0.99
	C2	0.96	0.98	0.97
	C3	0.87	0.89	0.88
DenseNet201 model	C0	0.96	0.89	0.92
	C1	0.99	0.99	0.99
	C2	0.98	0.99	0.99
	C3	0.81	0.96	0.93
Proposed model	C0	0.98	0.99	0.99
	C1	1.00	1.00	1.00
	C2	0.98	1.00	0.99
	C3	0.99	0.98	0.99

**Fig. 6:**
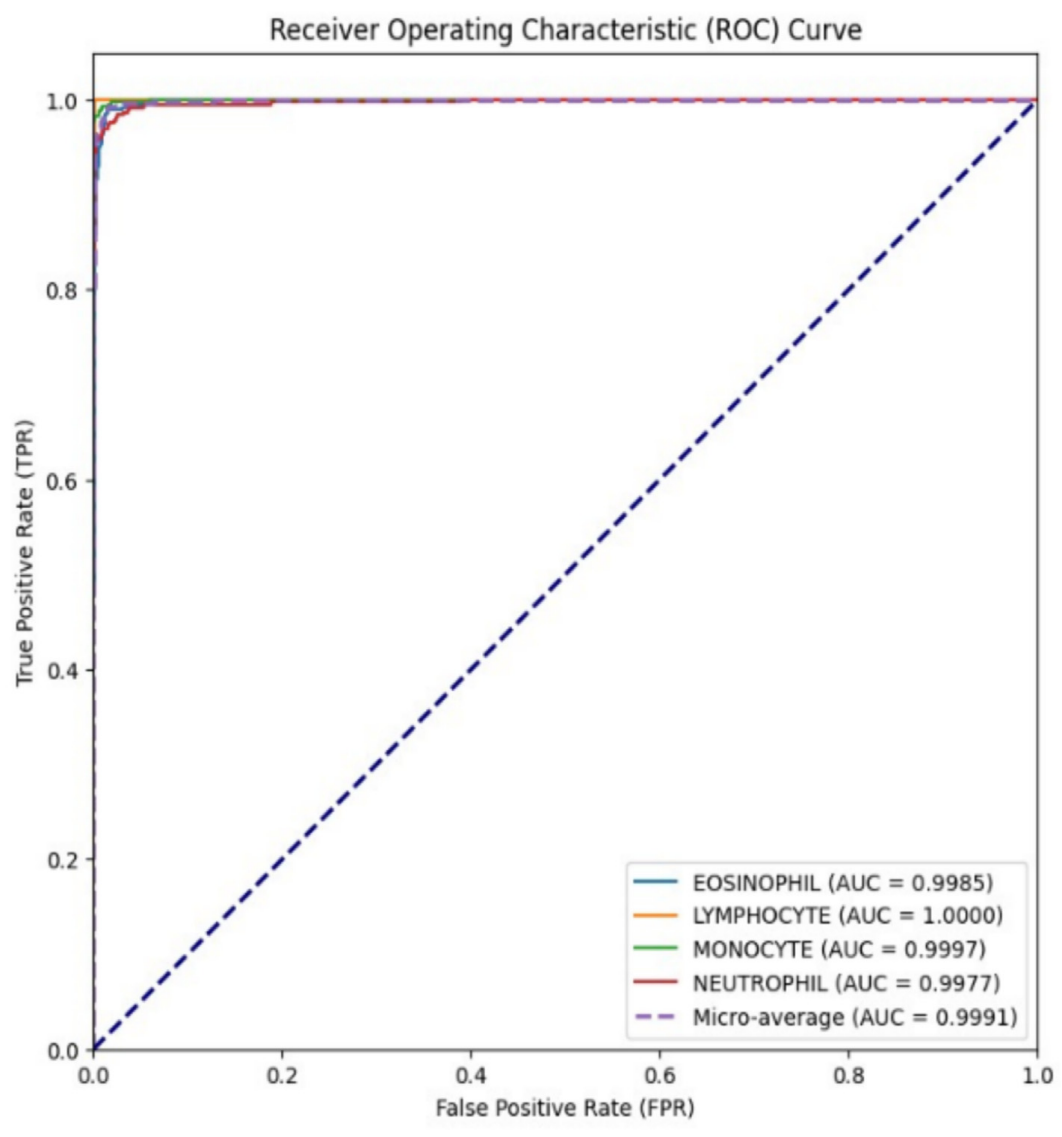
Applied model ROC-AUC curve.

Fig. 6 shows the receiver operating characteristic-area under the curve (ROC-AUC) curve for proposed model, the ROC study demonstrates that has high AUC values, indicating its extraordinary discriminating power across various cell types. The ‘LYMPHOCYTE’ and ‘EOSINOPHIL’ classes, in particular, obtained flawless AUC ratings of 1, emphasizing the model's exceptional capacity to correctly identify these cell types. The macro-average AUC overall 0.9991 indicates that all classes consistently perform exceptionally well in terms of categorization. These results place the suggested model in a strong and promising position for classifying cell types.

**Fig. 7:**
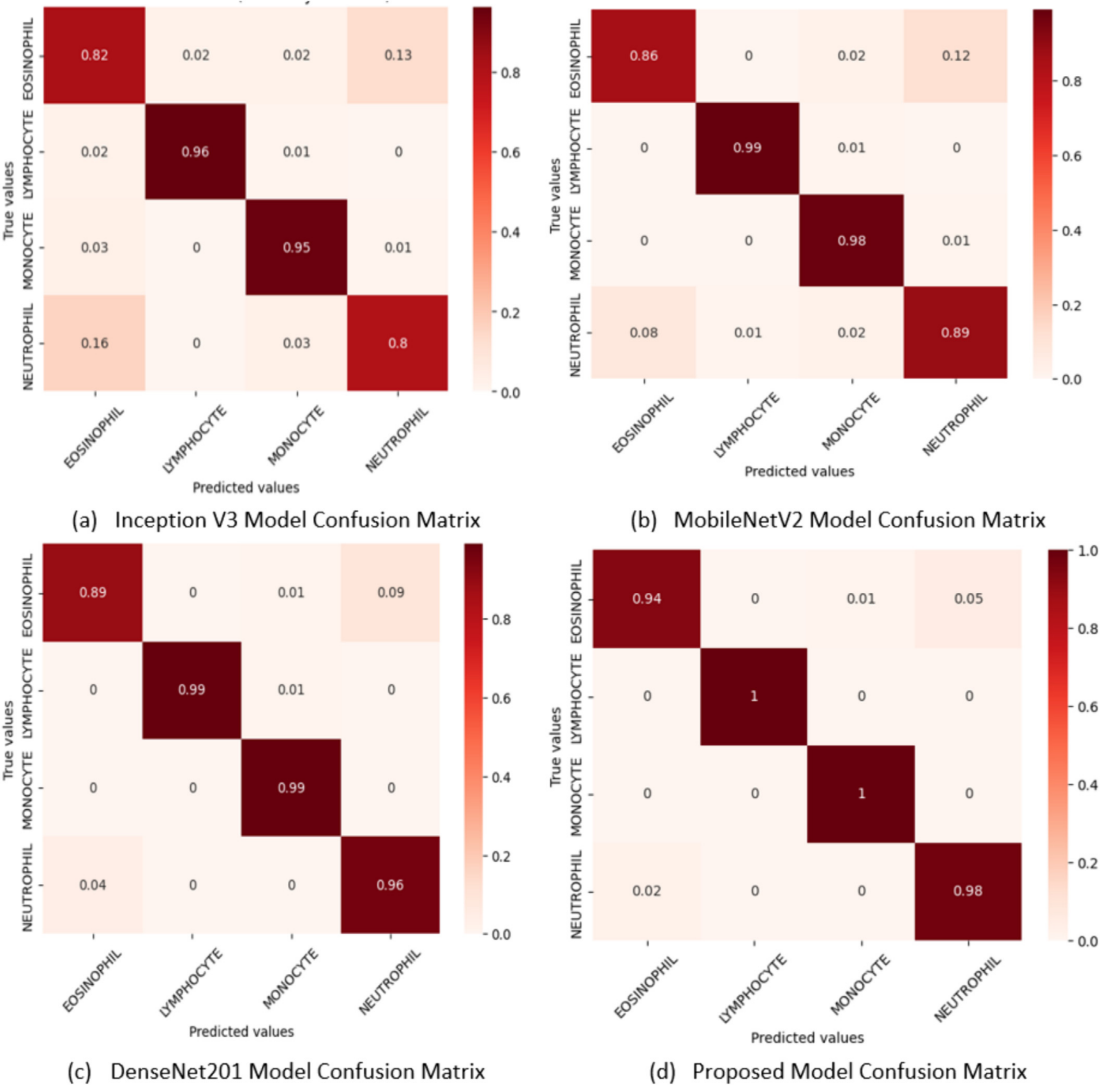
Confusion matrix results for all applied model.

Fig. 7 shows the all-confusion matrices where all model scores are generated. In the confusion matrix, rows indicate actual test image levels, whereas columns represent the predicted levels of test images. True-positive values are represented diagonally. It is evident that the confusion matrix of the proposed model shows a significant dominance along the diagonal, suggesting that it has a higher rate of accurate classifications across all classes compared to the other models. The proposed model is evidently highly accurate in distinguishing between various classes and minimizing misclassifications. Overall, the comparative result of the confusion matrix demonstrates the exceptional accuracy of the proposed model in classifying instances across multiple compared to Inception V3, MobileNetV2, and DenseNet201.

**Fig. 8:**
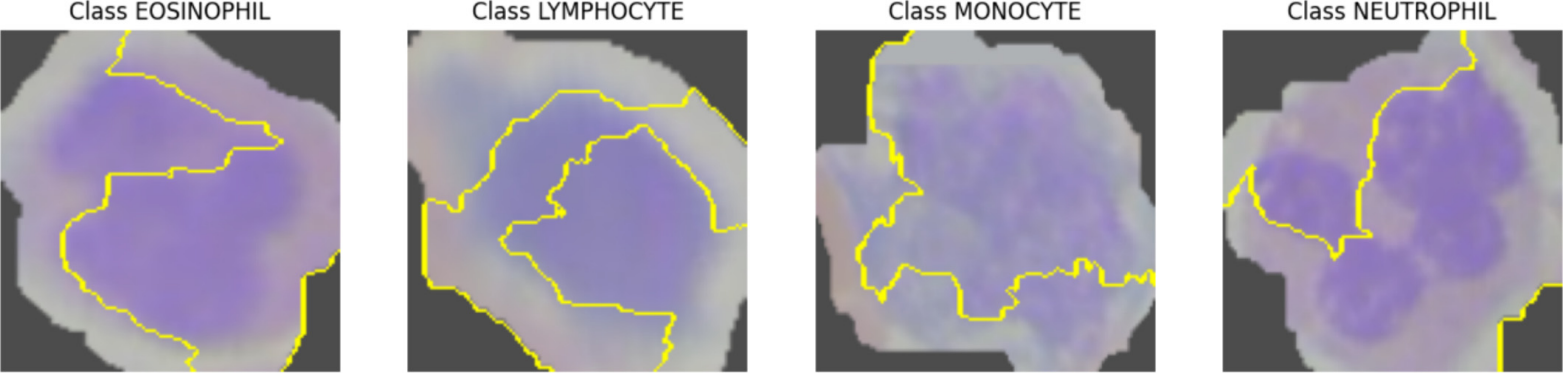
Saliency map for predicted image class.

In Fig. 8, we employed an XAI technology called LIME, which employs a local, understandable model to approximate any black box ML architecture and offer a justification for each prediction. It generated image segmentations and emphasized the crucial areas for categorization.^41^

Fig. 8 draw attention to the areas of the provided image that significantly affect the predicted outcome of the model for a given class. Most of the images showed the important regions of the model. To help with interpretation, the yellow boundaries or lines indicate which areas of the image are most important in influencing the forecast made by the model for a given class. This facilitates comprehension of the local decision-making process of the model for specific instances.

**Fig. 9:**
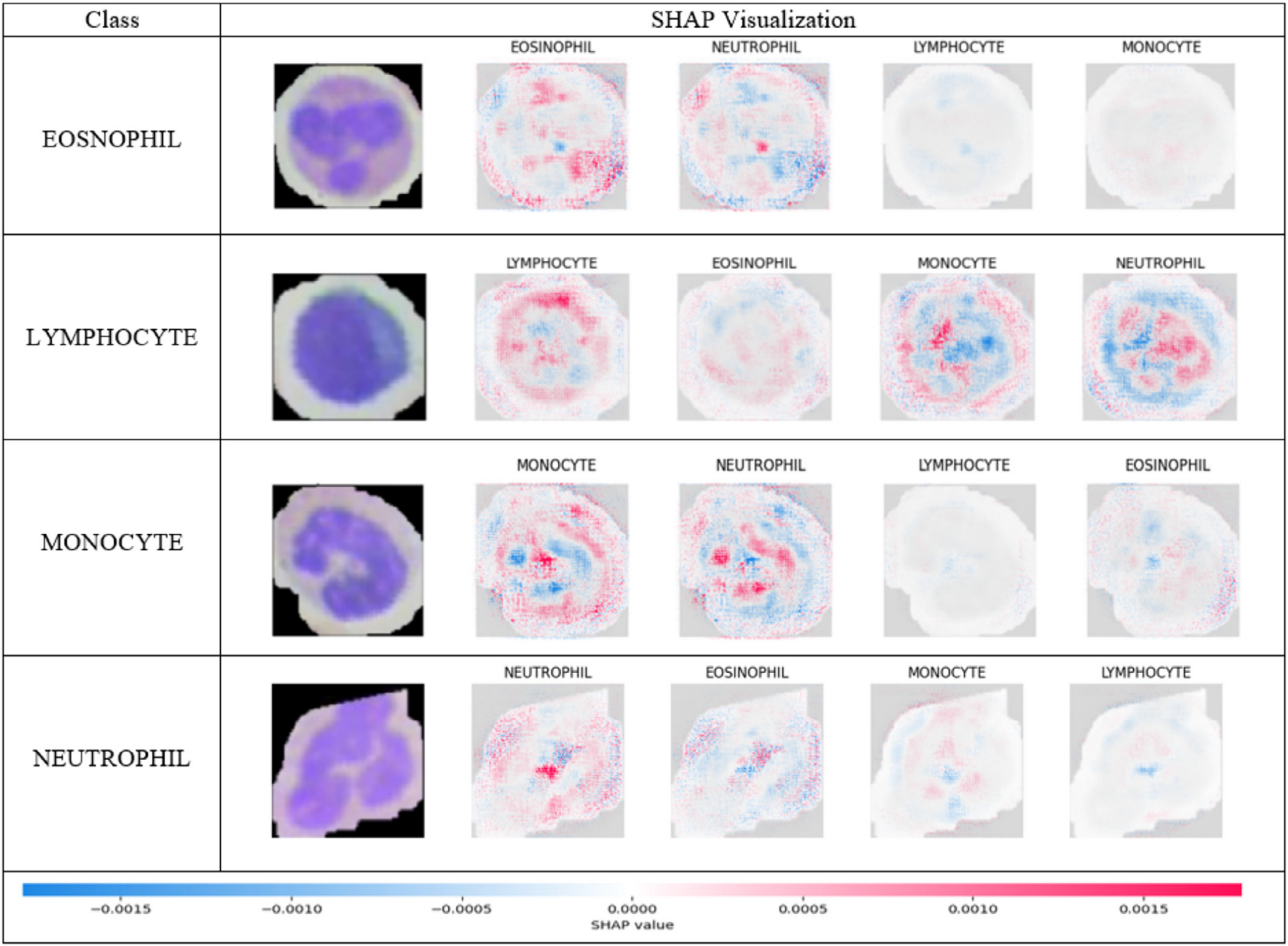
Saliency map for four classes using SHAP explainer.

By giving feature relevance ratings to specific pixels in the picture, SHAP offered a more precise account of the model's predictions. SHAP identify most important portion in the image where pink part explain significance of image that are correctly identified and blue area are normal area.^42^ We visualized the SHAP scores to demonstrate which portions of the image contributed the most to the model's decision. These graphics can help medical practitioners understand the model's predictions and offer insightful information about the model's decision-making process. Fig. 9. shows the saliency map for four class SHAP values.

The visual representations include SHAP values, with blue pixels representing negative impacts and red pixels showing positive contributions to the model's predictions. These depictions highlight the regions inside the image that significantly affect the model's decisions, providing deep insights into its reasoning processes and assisting medical professionals in the interpretation. Fig. 9 displays the SHAP results for a specific image, showcasing explanatory visuals for four classes including eosinophils, lymphocytes, monocytes, and neutrophils. From the initial row, it is clear that the first image explanation shows a higher number of red pixels, suggesting the presence of eosinophils in the cell. However, there is a noticeable absence of red pixels in lymphocytes, monocytes, and neutrophils. Hence, the SHAP explanation images indicate that the input image does not depict lymphocytes, monocytes, and neutrophils. In the second row, the SHAP explanation images for eosinophils, monocytes, and neutrophils lack red pixels and have a high number of blue pixels. However, the SHAP explanation image for lymphocytes has a noticeable number of red pixels. The image corresponds to lymphocytes. In the third row, the SHAP explanation image for monocytes displays a significant number of red pixels, whereas the fourth row's SHAP explanation image for neutrophils exhibits a noticeable concentration of red pixels.

**Fig. 10:**
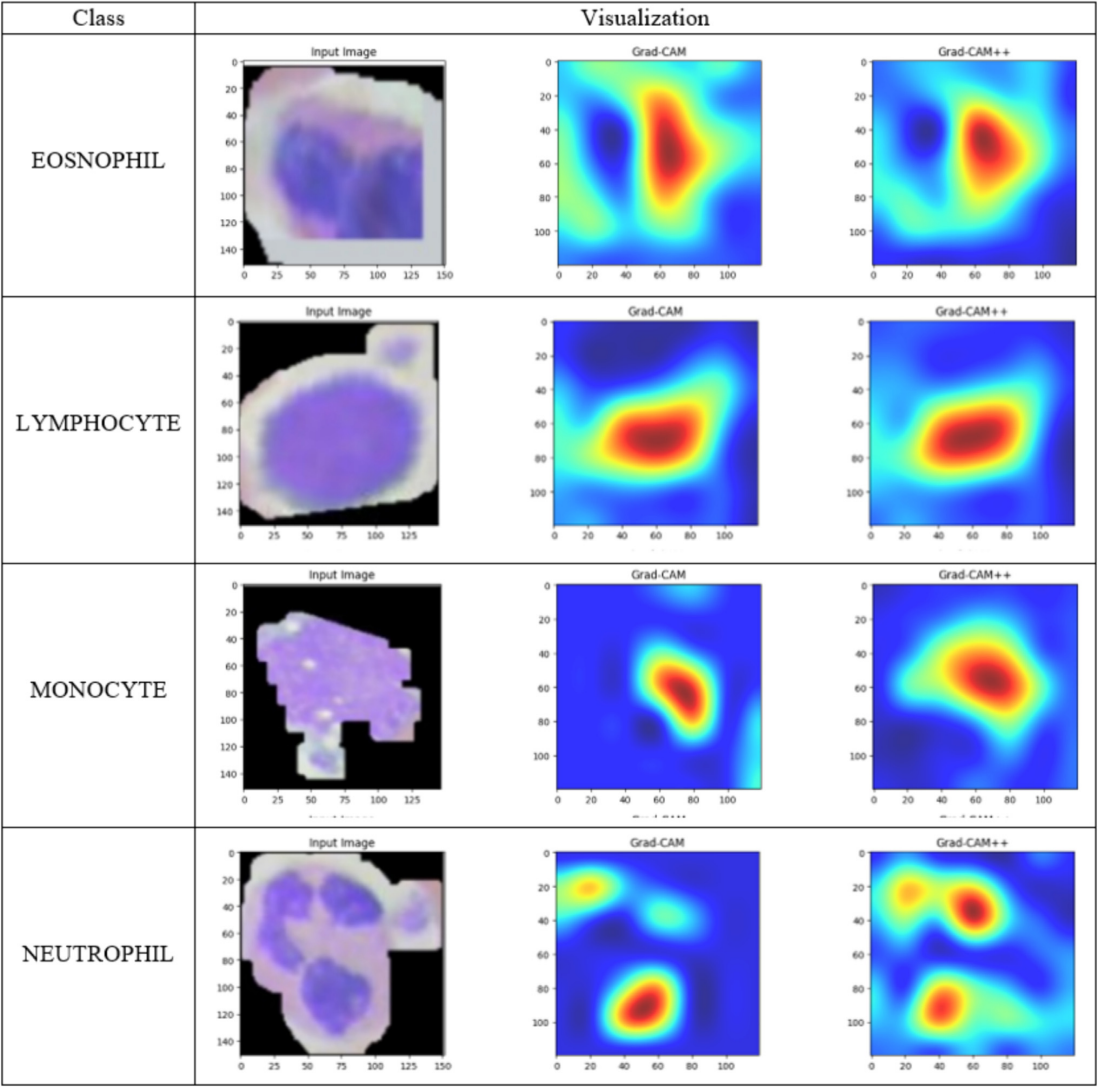
Class-wise Grad-CAM and Grad-CAM++ visualization.

Fig. 10 shows the Grad-CAM approach where Grad-CAM and Grad-CAM++ gives a better understanding of our proposed model. Grad-CAM leverages gradient information from the convolutional final layer to assess the impact of each neuron on decision-making. The ‘Input Image’ column displays original blood cell images categorized as eosinophils, lymphocytes, monocytes, and neutrophils. The Grad-CAM column superimposes heatmaps onto these images, emphasizing the regions that are crucial for disease detection according to the model presented. Warmer colors (red and yellow) signify higher importance, whereas cooler colors (blue) signify lower importance. Based on the Grad-CAM++ along with Grad-CAM analyses, the specified spot of the eosinophils image shows a higher level of red heatmap. This is represented by the corresponding heatmap presented in Fig. 10. The central region of the image has the most significant impact on the classification of lymphocytes, as indicated by the heatmap centered accordingly. On the other hand, in the case of monocytes, the right portion of the image has a higher intensity of red in the heatmap, and the corresponding heatmap has been displayed accordingly.

The output above showcases the application of XAI techniques, specifically Grad-CAM and Grad-CAM++, which offer transparent and understandable predictions. The specific predictions made in this model are easily understandable for both patients and medical professionals, even if they are not familiar with the underlying model. Highlighting important features through Grad-CAM and Grad-CAM++ provides individuals with useful insights into the model's decision-making. Our proposed model not only achieves high accuracy but also stands out in its ability to provide detailed explanations for specific classifications.

Table 7 shows a selection of current studies on blood cell identification that are organized by architecture, year of publication, number of classes, and performance evaluation with our study. Various CNN models, including TL-based models and custom CNN, were used to classify the detection of blood cells. Furthermore, our work has developed a streamlined and effective model that outperforms previous studies in terms of accuracy while also addressing the constraints associated with other elements. Moreover, the present work has implemented the most effective model in a web-based application designed to classify blood cell subtypes.

**Table 7:** Comparisons of the results with relevant research.

Reference	Publishing year	Dataset amount	Class used	Applied architecture	Best architecture with accuracy	Explainable AI used and deployment	Limitation
Zhu et al.^6^	2023	12,500	4	CNN-AdaboostM1, Xception-LSTM, DLBCNet	DLBCNet 95.05	No	Accuracy low.
Tausneem et al.^8^	2023	–	2	DCAE-CNN	97%	No	Used single model, Limited study in classification.
Sarang Sharma et al.^9^	2022	12,444	4	DenseNet121	98.84%	No	Used single model
Meenakshi et al.^14^	2022	12,500	4	AlexNet, GoogLeNet, ResNet50 Custom CNN	Custom CNN with SGD (94.25%)	No	Lower accuracy
Erdal Başaran's^15^	2022	12,435	4	SqueezeNet	95.88%	No	Lower accuracy, Used single model.
Yao et al.^16^	2021	11,865	6	VGG16, VGG19, Inception-V3, ResNet-50, TWOD-CNN	TWOD-CNN (91.6%)	No	Lower size of data.
Yao et al.^17^	2021	364	4	RCNN and Yolov4	RCNN (96.25%)	No	Lower size of data.
Yusuf Yargı Baydilli and Ümit Atila's^20^	2020	4000–11,000	4	Capsule networks	96.86%	No	Lower size of data.
Shahin et al.^23^	2019	2551	5	WBCsNet	96.1%	No	Lower size of data.
Our proposed approach	–	12,444	4	Inception V3, MobileNetV2, DenseNet201 and Proposed model	Proposed model (99.12%)	Used Grad CAM, Grad CAM++, LIME, SHAP, Android and Web	–

### Implementation

**Fig. 11:**
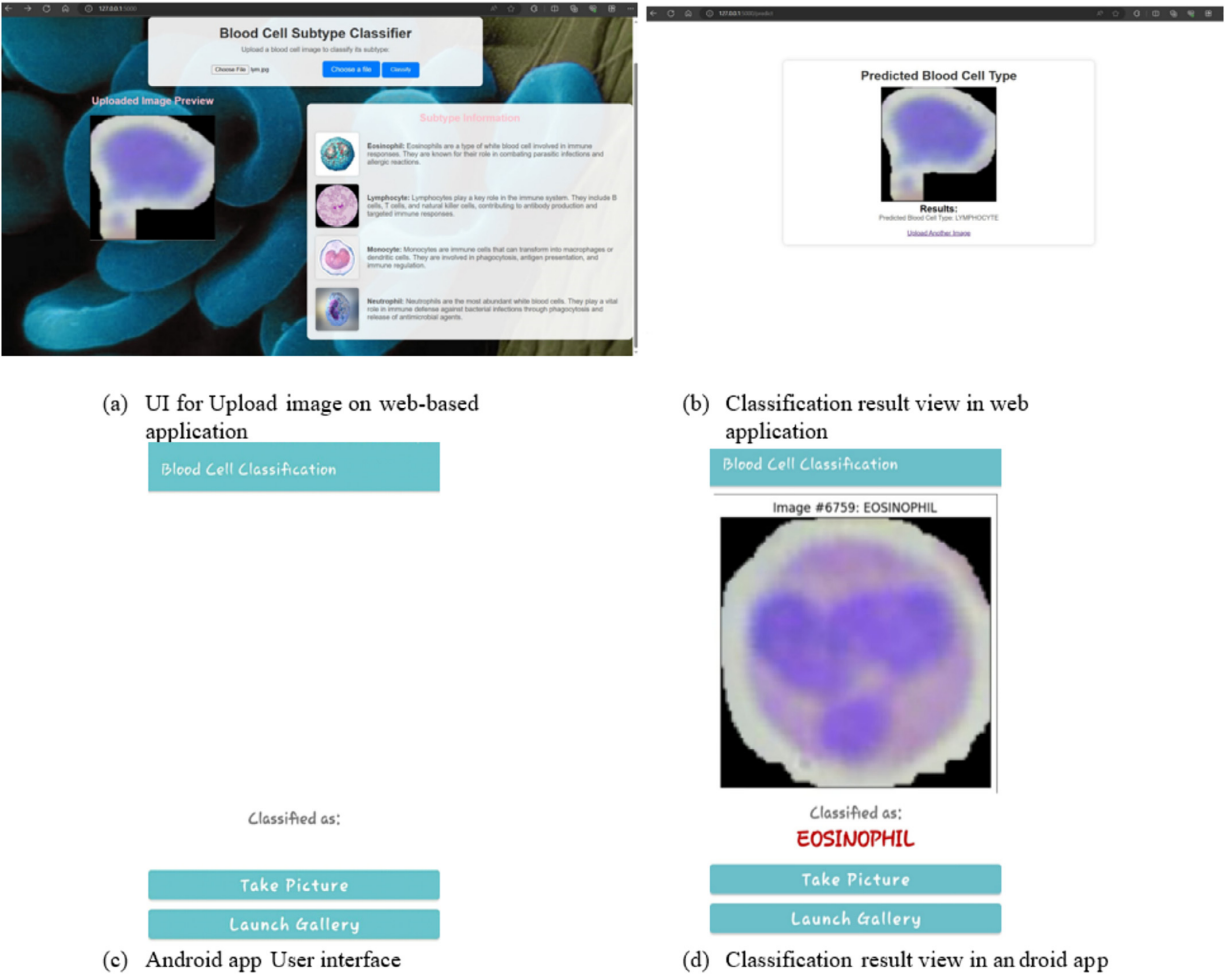
Blood cell detection application.^43^

The deployment of a DL model refers to the way of integrating a fully developed DL model into a setting for production, enabling its utilization for its designated objectives. The ultimate goal of blood cell categorization, detection, and research is to provide societal benefits and fulfill the needs of patients. Rapid identification and categorization of blood cells in the early stages of illness are of utmost importance in facilitating prompt diagnosis and treatment. This enables healthcare professionals to implement appropriate medical treatments, ultimately leading to superior patient outcomes. Therefore, accurate disease identification plays a crucial role in addressing this issue. The implementation of a comprehensive system for the classification of blood cells equips doctors, medical researchers, and healthcare providers with effective and precise resources, thereby augmenting their capacity to diagnose illnesses, track patient well-being, and engage in advanced medical investigations to improve patient treatment and gain valuable medical knowledge. The deployment of the model has been considered for integration into applications that are both web- and Android-based within a complete system. Fig. 11(a) and (b) depicts the process of deploying the blood cell classification system locally, as well as the outcome of the deployed model in predicting the class of blood cells. Fig. 11(c) and (d) shows the Android application's graphical user interface and the results of the real-time classification.

In this work, we have utilized the most successful model to predict blood cell categorization using image analysis. The web-based system was created by the current research using a wide variety of Python modules and auxiliary technologies. Cascading style sheets and HTML were used to construct the front-end design of the website. The backend infrastructure has been implemented using the Flask framework, which is based on the Python programming language. Users have the capability to submit an image depicting a blood cell. Subsequently, this image undergoes classification by the proposed model. The outcome of this classification is displayed on the screen.

Extensible Markup Languagewas used to construct the user interface of an Android application, whereas Java was used to implement the backend functionality. The most efficient model for image classification has been converted into a “tflite” version of TensorFlow, which has been made more effective. The lightweight TensorFlow model file has been integrated into the Android application using Java programming. The developed Android application works with all versions of Android, from 5.0 to 12.0. It allows users to identify blood cells by capturing real-time images or selecting existing images from their devices. This research has enabled open access to our Android application, designed specifically for smartphones, through a publicly accessible GitHub repository.^43^ The mentioned model is characterized by its low weight and the ability to rapidly and accurately predict outcomes while providing corresponding findings.

## Conclusion

The study effectively demonstrated the potential of an effective CNN in accurately classifying WBCs, which play an important part in the human immunity system. We employed advanced image pre-processing techniques such as padding, thresholding, erosion, dilation, and masking to reduce noise, enhance feature visibility, and identify the relevant regions, thereby facilitating more accurate classification. In various performance metrics, our proposed model surpassed existing transfer learning models, such as Inception V3, MobileNetV2, and DenseNet201. It achieved a testing accuracy of 99.12%, a precision of 99%, and an F1 score of 99%. In addition, the use of SHAP and LIME for model interpretability has offered a more profound understanding of the decision-making process of the proposed model, fostering trust and transparency among medical professionals. The Grad-CAM and Grad-CAM++ developments have enhanced this by providing class-discriminative localization capabilities. Grad-CAM++ has a slight advantage over Grad-CAM in accurately identifying predicted areas. In conclusion, incorporating our model into a comprehensive system accessible through web and Android platforms greatly empowers medical professionals to classify blood cells with exceptional precision and effectiveness. In the future, we plan to expand our work to address clinical-grade identification of additional cell types, such as platelets and red blood cells.

## Declaration of competing interest

The authors declare that they have no known competing financial interests or personal relationships that could have appeared to influence the work reported in this article.
